# Chloride Ion Adsorption by Modified Pisha Sandstone-Based Cementitious Materials

**DOI:** 10.3390/gels12070587

**Published:** 2026-07-02

**Authors:** Changming Li, Shuxian Lu, Shunbo Zhao, Xinxin Ding, Weihua Li, Jingyuan Zhao, Xianglin Xu, Wenbin Xu

**Affiliations:** 1 International Joint Research Lab for Eco-Building Materials and Engineering of Henan, North China University of Water Resources and Electric Power, Zhengzhou 450045, China; lichangmingh@163.com (C.L.); 17513115224@163.com (S.L.); dingxinxin@ncwu.edu.cn (X.D.); 13839011086@163.com (J.Z.); 2 Xuchang Innovation Center of Low-Carbon and Eco-Building Materials Technology, Zhongyuan Institute of Science and Technology, Zhengzhou 450042, China; 3 Shuang Ta Paint Technology Co., Ltd., Kaifeng 475000, China; 18625536172@163.com (X.X.); xuwe.nbin@163.com (W.X.)

**Keywords:** modified Pisha sandstone, chloride ion adsorption, adsorption performance, low cost, eco-friendly

## Abstract

Pisha sandstone (PS) has potential as a low-cost adsorbent due to its abundant surface-active adsorption sites. In this work, mechanical grinding coupled with high-temperature calcination was employed to activate and modify raw PS for improved chloride ion adsorption performance and efficient resource utilization. Adsorption kinetic experiments demonstrated that the PS modified via 15 min of mechanical grinding (PSM15) exhibited the optimal chloride adsorption performance and achieved adsorption equilibrium within 240 min. The adsorption kinetics data were well fitted by the pseudo-second-order model, indicating that chemisorption dominates the chloride adsorption process. The chloride removal efficiency of PSM15 reached a maximum value of 33.3%, which was superior to that of calcined PS (30.1%) and raw PS (23.6%). Combined characterization results from XRD, FTIR, and SEM-EDS revealed that mechanochemical activation does not alter the main crystalline phases of the material. Instead, it significantly enhances chloride adsorption capacity by refining crystallite size, exfoliating layered microstructure, and exposing surface active sites. Moreover, the in situ formation of C–S–H gel reinforces chloride immobilization via physical encapsulation and electrostatic attraction. Collectively, the enhanced chloride adsorption by modified PS can be attributed to synergistic mechanochemical activation, surface and interlayer retention, and gel-mediated immobilization. As a low-cost and eco-friendly adsorbent, the PS-based cementitious material shows promising application potential in chloride-containing wastewater purification.

## 1. Introduction

In global infrastructure construction and service safety, chloride-induced degradation has become one of the primary threats to the durability of concrete structures [1,2].Chloride ions are highly mobile and aggressive. They can penetrate concrete and disrupt the passive film on the surface of steel reinforcement, thereby initiating reinforcement corrosion. This process can lead to concrete cracking, reduced load-bearing capacity, and a substantially shortened service life, resulting in considerable economic losses and serious safety risks. In addition, chloride ions are representative anionic contaminants in saline wastewater treatment. Their high mobility and low immobilization efficiency place more stringent requirements on the ion removal and retention capacities of water treatment materials. Therefore, selecting chloride ions as the target adsorbate reflects the chloride-binding capacity of a material in chloride attack environments and helps evaluate its applicability for the treatment of chloride-containing wastewater [3]. Driven by the increasingly stringent decarbonization policies implemented in the building materials sector by major economies worldwide [4,5], traditional high-carbon cementitious materials face an urgent need for a green and low-carbon transition. The development of novel low-carbon cementitious materials with excellent chloride ion binding capacity is therefore of critical importance for improving the durability of concrete structures and mitigating chloride-induced degradation [6].

Geopolymers, a new class of green cementitious materials produced by alkali activation of aluminosilicate precursors, have attracted considerable attention in civil engineering and pollution control owing to their low carbon footprint, high strength, superior durability, and excellent ion immobilization capacity [7,8,9]. Their three-dimensional network gel structure can stably retain chloride ions through physical encapsulation, chemical bonding, and electrostatic adsorption, making them an ideal low-carbon carrier for chloride ion binding [10]. PS, an abundantly available aluminosilicate waste material in the Shanxi–Shaanxi–Inner Mongolia border region, is rich in minerals such as quartz, feldspar, and montmorillonite [11,12]. This composition offers inherent advantages for geopolymer synthesis. Moreover, PS possesses a distinctive layered structure and a certain degree of cation exchange capacity. Converting PS into high-performance geopolymer cementitious materials therefore represents a promising strategy for solid waste valorization, ecological restoration, and high-value utilization [13]. Nevertheless, the intrinsic anion adsorption capacity of PS is relatively weak, limiting the effectiveness of its direct application [14]. Consequently, mechanochemical activation was employed to modify PS, aiming to improve its surface physicochemical properties and enhance the density of positively charged active sites. This modification is intended to enhance the exchange of chloride ions.

To address chloride ion contamination, common approaches include chemical precipitation, membrane separation, electrochemical methods [15], and adsorption [16]. However, these techniques generally suffer from drawbacks such as high operating costs, substantial energy consumption, secondary pollution risks, and limited ion selectivity. In contrast, adsorption is regarded as one of the most promising water treatment technologies due to its operational simplicity and low cost. Adsorption relies on physical or chemical interactions between the adsorbent and chloride ions to achieve efficient separation. To date, research on the preparation and modification of PS-based geopolymer materials remains relatively limited. The chloride ion binding performance, kinetic behavior, and underlying microscopic mechanisms of these materials in chloride-rich environments are still not well understood. Therefore, a systematic investigation into the chloride ion adsorption behavior of PS is of great significance for expanding its application in chloride-containing wastewater treatment [17].

Meanwhile, layered double hydroxides (LDHs) and cementitious systems have also attracted considerable attention in the field of chloride ion immobilization. Zhang et al. [18] prepared Mg/Al–CLDH via high-temperature calcination. The material displayed excellent chloride ion adsorption capacity, governed primarily by ion exchange and the structural memory effect, and thus represents an efficient adsorbent for water dechlorination. Li et al. [19] investigated the physical adsorption mechanism of chloride ions at the C–S–H gel interface, along with relevant characterization methods and the influence on chloride ion transport. Their work provides important theoretical support for elucidating the chloride ion binding mechanisms in cement-based materials. Xu et al. [20] synthesized CaMnFe–LDHs via a coprecipitation method. The incorporation of these materials into cement pastes significantly improved chloride ion binding performance, indicating considerable potential for mitigating chloride-induced deterioration. Mechanical grinding and high-temperature calcination are two common methods for enhancing the reactivity of clay minerals. The former refines particles through mechanochemical effects and induces lattice defects and surface bond breaks, thereby increasing the surface energy of the minerals; the latter promotes dehydroxylation of layered silicate minerals through thermal effects, causing a partial phase transition to an amorphous state, which significantly increases the anion exchange capacity. Both activation methods effectively break down the inert structure of minerals, exposing more active adsorption sites.

Existing studies indicate that appropriate modification can substantially improve the adsorption performance of PS. Wang et al. [21] modified PS with sodium ions and found that Na-PS exhibited significantly higher adsorption capacities for Cd(II) and Cu(II) than pristine PS, demonstrating promising potential for heavy metal remediation. Further modification with aluminum (Al) markedly enhanced the phosphate adsorption capacity of PS. Electrostatic attraction dominated the adsorption mechanism, and the resulting Al–PS material showed dual applicability in wastewater treatment and soil amendment [22].

This study used PS as the precursor for adsorbent preparation and chloride ions as the target adsorbate. The effects of various modification methods and processing parameters on chloride adsorption performance were evaluated to determine the optimal treatment conditions. Adsorption kinetic models were employed to describe chloride ion adsorption on PS and determine the dominant rate-controlling mechanism. The underlying adsorption mechanism was further clarified through XRD, FTIR, and SEM–EDS. These analyses revealed variations in phase composition, functional groups, microstructure, and elemental distribution. Based on these results, modified PS adsorbents were prepared, and their adsorption kinetics and key influencing factors were systematically examined. Ultimately, this work aimed to convert PS from an environmental burden into a high-efficiency adsorbent and to provide a viable material for the treatment of chloride-contaminated wastewater.

## 2. Results and Discussion

### 2.1. Adsorption Kinetics of Chloride Ions on Modified PS

**Figure 1:**
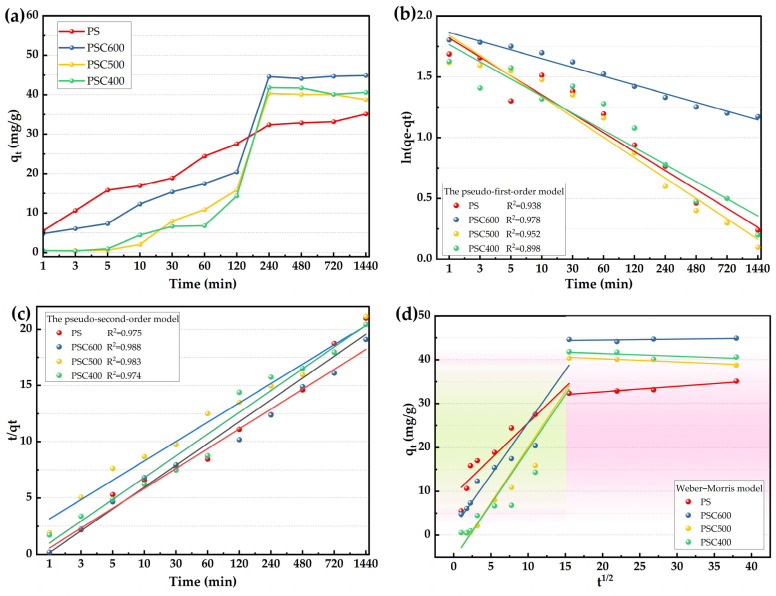
(**a**) Adsorption kinetics of chloride ions on high-temperature calcined PS. (**b**) Fitting using a first-order model, (**c**) a second-order model, and (**d**) a Weber–Morris model to describe the adsorption kinetics of high-temperature calcined PS. Sample designation of modified PS materials. PSCx denotes samples prepared at a calcination temperature of x (°C).

**Figure 2:**
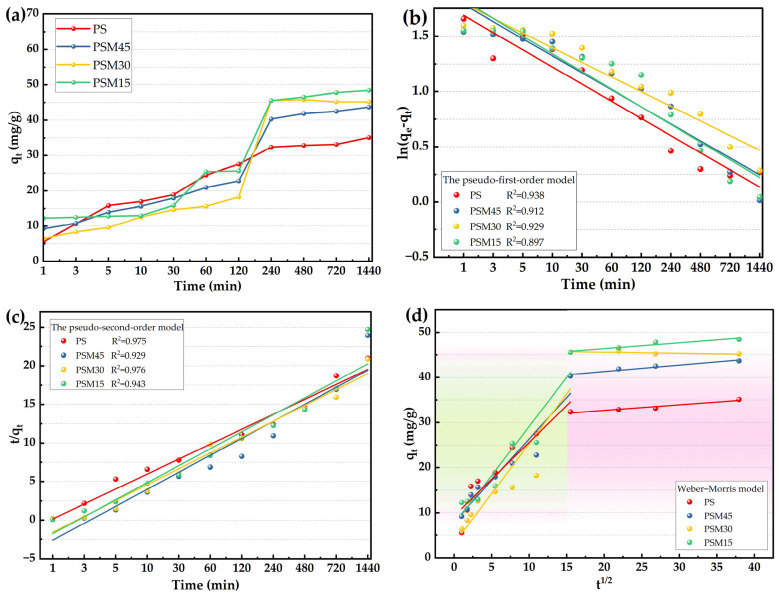
(**a**) Adsorption kinetics of chloride ions on mechanically ground PS. (**b**) Fitting using a first-order model, (**c**) a second-order model, and (**d**) a Weber–Morris model to describe the adsorption kinetics of mechanically ground PS. Sample designation of modified PS materials. PSMy denotes samples prepared at a mechanical grinding time of y (min).

At an initial chloride ion concentration of 0.01 mol/L (355 mg/L), Figure 1 shows the adsorption kinetics of chloride ions onto high-temperature calcined PS (PSC), and Figure 2 shows the corresponding kinetics onto mechanically ground PS (PSM). During the initial stage (0–120 min), the adsorption capacity increased rapidly, indicating a fast adsorption process. This behavior can be attributed to the high initial concentration of chloride ions and the abundance of unoccupied active sites on the surface of modified PS, which promoted rapid mass transfer and efficient chloride ion capture. During the intermediate stage (120–240 min), the adsorption process entered a transitional regime [21]. This result suggests that, in addition to surface adsorption, intraparticle diffusion gradually became dominant. The internal pores and interlayer domains provided newly accessible active sites, thereby enhancing chloride ion uptake. After 240 min, the adsorption rate decreased markedly, and the system progressively approached dynamic equilibrium. At this stage, most active sites had been occupied, and the mass transfer driving force was reduced. Consequently, the adsorption process was mainly controlled by diffusion until equilibrium was reached [23].

As shown in Figure 1a and Figure 2a, after high-temperature calcination, the equilibrium adsorption capacity of PS for chloride ions increased with elevated calcination temperature. This enhancement may be attributed to the dehydroxylation and lattice rearrangement of clay minerals, such as montmorillonite and illite, during calcination. These processes improved the surface activity of the material and introduced additional active sites for chloride ion adsorption. The specific surface area measurements also indicated that the original PS had a specific surface area of only 0.36 m^2^/g. After calcination at 400 °C, 500 °C, and 600 °C, the values increased to 0.40 m^2^/g, 0.48 m^2^/g, and 0.51 m^2^/g, respectively. This finding indicates that appropriate heat treatment can improve the pore structure and surface properties of the material. Among the calcined samples, the sample treated at 600 °C exhibited the most pronounced modification effect, and no obvious excessive sintering was observed.

After mechanochemical grinding modification, the adsorption capacity of PS initially increased and subsequently decreased with increasing grinding time. Moderate grinding can refine particles, increase the specific surface area, and partially disrupt the mineral lattice through mechanochemical effects. This process exposes more exchangeable sites and enhances the chloride ion adsorption capacity. The specific surface area of PSM15 increased to 0.87 m^2^/g, indicating that short-duration grinding substantially promoted particle surface exposure. However, when the grinding time was further extended to 30 min and 45 min, the specific surface areas decreased to 0.67 m^2^/g and 0.59 m^2^/g, respectively. This reduction suggests that excessive grinding can readily induce agglomeration of fine particles and collapse of the pore structure. As a result, the number of effective adsorption sites was reduced, ultimately leading to a reduction in adsorption capacity [22,24].

To clarify the chloride adsorption mechanism, the kinetic data were fitted using the pseudo-first-order, pseudo-second-order, Weber–Morris intraparticle diffusion, and Elovich models. Table 1 summarizes the fitting parameters obtained from the pseudo-first-order, pseudo-second-order, Weber–Morris intraparticle diffusion, and Elovich models for chloride adsorption on different PS-based adsorbents. As shown in Figure 1 and Figure 2, the pseudo-second-order model (R^2^ up to 0.988 in Figure 1c and 0.976 in Figure 2c) generally provided better fitting performance than the pseudo-first-order model, indicating that surface interactions and active site occupation played important roles in chloride uptake. The pseudo-first-order model showed relatively high (R^2^) values for some samples, but the calculated equilibrium adsorption capacities were much lower than the experimental values, suggesting that this model could not adequately describe the whole adsorption process. The Weber–Morris fitting curves shown in Figure 1d and Figure 2d exhibit two distinct linear stages and do not pass through the origin, demonstrating that chloride adsorption was jointly affected by boundary layer mass transfer and intraparticle diffusion rather than being controlled only by intraparticle diffusion. The first stage corresponded to rapid chloride migration to the external surface and accessible pore regions, whereas the second stage reflected slower diffusion and a gradual approach to equilibrium. In addition, the Elovich model showed moderate to good fitting performance, indicating the presence of heterogeneous surface adsorption characteristics. Overall, the chloride adsorption process on modified PS can be considered to involve chemisorption contributions or surface chemical interactions.

In summary, considering both removal cost and efficiency, PSC600 and PSM15 were selected for subsequent ion adsorption tests to further compare their adsorption capabilities.

### 2.2. Evaluation of Chloride Ion Removal Efficiency Based on Modification Methods

**Figure 3:**
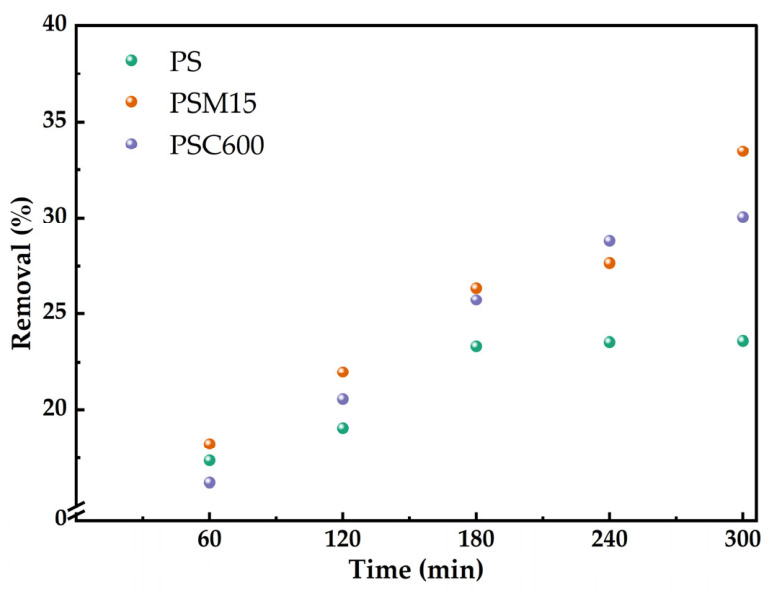
Chloride ion removal efficiency of the PS, PSC600 and PSM15.

Figure 3 presents the percentage removal of chloride ions by raw PS, PS modified by mechanical grinding for 15 min (PSM15), and PS modified by calcination at 600 °C (PSC600). During the initial 60 min, all three materials exhibited relatively rapid chloride ion adsorption rates. In this period, PSM15, PSC600, and PS removed approximately 18.2%, 16.5%, and 17.3% of the chloride ions, respectively. As the contact time increased, the adsorption rate gradually declined. This deceleration is attributed to the progressive saturation of surface adsorption sites and the densification of cementitious products. Nevertheless, sustained chemical binding and physical encapsulation contributed to a slow but continuous increase in removal efficiency, which had not yet reached equilibrium under the experimental conditions. At the final sampling point, the chloride ion removal efficiencies of PSM15, PSC600, and PS were approximately 33.3%, 30.1%, and 23.6%, respectively. Under the given experimental conditions, PSM15 exhibited the highest chloride ion removal capacity, followed by PSC600, while raw PS showed the lowest. These results demonstrate that both mechanical grinding and high-temperature calcination effectively enhance the chloride ion adsorption performance of PS, with PSM15 proving to be more pronounced.

This result may be attributed to the fact that mechanical grinding treatment not only increases the specific surface area of the material and improves the mass transfer conditions but also activates the particle surfaces through mechanical grinding, resulting in a significant increase in the specific surface area [25]. This provides a large number of active sites for chloride ion adsorption and significantly increases the effective contact area between chloride ions and the surface of the argillite. In contrast, although high-temperature calcination can activate mineral constituents, partial agglomeration of active components induced by thermal effects may occur [26], limiting the gains in specific surface area and active sites relative to grinding. Consequently, the chloride ion removal efficiency followed the order PSM15 > PSC600 > PS, indicating that mechanical grinding represents an effective physical modification strategy for enhancing the chloride ion adsorption capacity of PS. Previous studies have reported that natural or modified clay-based materials, including montmorillonite and bentonite, typically exhibit negatively charged surfaces [27]. Consequently, their adsorption capacity for anions is generally limited in the absence of surface modification or functionalization. In most studies, unmodified clay materials exhibit low removal efficiencies for anions. In contrast, PSM15 in this study achieved a chloride removal efficiency of approximately 33%, demonstrating comparatively superior performance. This enhanced performance may be associated with structural changes induced by mechanochemical activation, which can increase the surface reactivity of the material. Based on these findings, PSM15 was selected for subsequent characterization experiments.

To further assess the chloride ion adsorption performance of the modified PS material, PSM15 was benchmarked against representative chloride adsorbents reported in recent studies, as summarized in Table 2.

### 2.3. Phase Composition Analysis (XRD)

**Figure 4:**
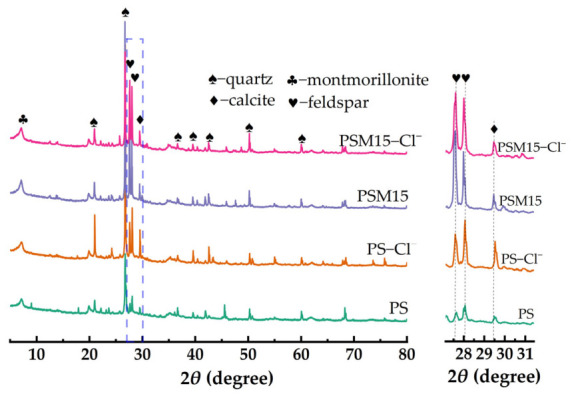
XRD patterns of mechanically ground PS material adsorbing chloride ions.

Figure 4 presents the XRD patterns of raw PS, PSM15, and the corresponding samples after chloride ion adsorption (PS–Cl^−^ and PSM15–Cl^−^). Comparative analysis reveals that the crystalline phase compositions of the four specimens are essentially identical, with quartz, montmorillonite, feldspar, and calcite being the dominant mineral phases. A comparison of the XRD patterns of PS and PSM15 reveals a marked increase in the intensity of characteristic diffraction peaks for minerals such as quartz and feldspar in PSM15, accompanied by sharper peak profiles. This observation indicates that mechanical grinding refines particle size and reduces agglomeration, thereby exposing more crystallographic planes while preserving the intrinsic crystal structure of quartz, thus achieving physical activation without structural degradation. The low-angle characteristic peak of montmorillonite at 2*θ* ≈ 5–8° became markedly intensified and narrowed after mechanical treatment. This variation can be attributed to improved structural ordering within the layered domains. Grinding can reduce particle size and eliminate associated impurities, thereby improving the coherence of the (001) crystal planes and increasing the diffraction intensity. This structural evolution may promote the migration of ions into interlayer channels, thereby enhancing adsorption performance [29]. In PSM15, the calcite characteristic peak at 2*θ* ≈ 29.4° appears more distinct, confirming that grinding breaks down mineral inclusions and strengthens secondary-phase diffraction signals.

Compared with PS, PS–Cl^−^ exhibits no discernible shifts in diffraction peak positions, peak broadening, or the emergence of new crystalline phases. Chloride ions are retained primarily through weak surface physisorption, with negligible interlayer ion exchange or chemical immobilization. Consequently, the crystal structure and interlayer order of the minerals remain largely unaffected. PS features coarse particles, a low specific surface area, and a scarcity of active sites, all of which contribute to its poor chloride ion adsorption capacity. The results showing the lowest PS removal rate in the chloride ion permeation test were consistent.

Compared with PSM15, the intensities of the quartz and feldspar characteristic peaks at 26–28° and 36–37° in PSM15–Cl^−^ are lower. This suggests that exchangeable cations in feldspar are leached by OH^−^ present in the pore solution, accompanied by localized hydrolysis of the aluminosilicate framework, which leads to partial disruption of the crystal structure and reduced crystallinity. Quartz subjected to mechanical grinding possesses a higher density of surface defects and enhanced reactivity, rendering it more susceptible to attack by OH^−^ and promoting dissolution of the surface layer [30]. Although such surface dissolution does not alter the bulk crystal structure of quartz, it diminishes the effective diffracting volume and generates an amorphous surface layer, thereby reducing the intensity of the corresponding diffraction peaks [31]. Chloride ions do not react directly with quartz or feldspar to form new phases. Nevertheless, the ingress of Cl^−^ displaces interlayer or surface-adsorbed OH^−^, alters the ionic equilibrium of the pore solution, and facilitates the continuous migration of OH^−^ toward the mineral surfaces, thereby sustaining a localized high-pH environment [32].

Mechanical grinding for 15 min significantly enhances the structural reactivity of PS toward chloride ions. The retained chloride ions are primarily incorporated into interlayer spaces or adsorbed onto defect sites, whereas raw PS only exhibits inert physisorption. Controlling the mechanochemical activation degree is essential for optimizing the chloride ion penetration resistance of modified PS.

### 2.4. Fourier Transform Infrared Spectroscopy Analysis (FTIR)

**Figure 5:**
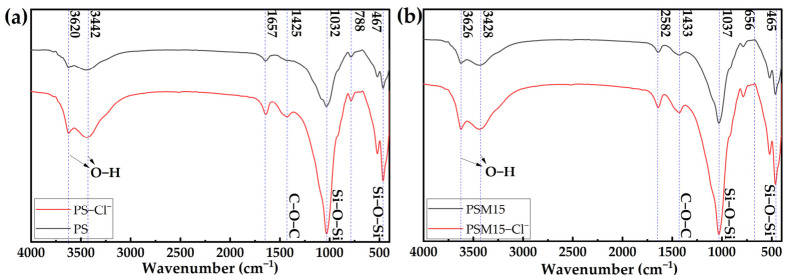
(**a**) PS and (**b**) PSM15—FTIR spectra before and after adsorption of chloride ions.

Figure 5 presents the FTIR spectra of PS and PSM15 before and after chloride ion adsorption. Figure 5a shows the FTIR spectra of PS and the chloride ion-adsorbed sample (PS–Cl^−^). In the spectrum of PS–Cl^−^, the O–H stretching vibration peaks at 3620 and 3442 cm^−1^ become sharper and more intense after chloride ion adsorption. This change indicates an alteration in the hydrogen bonding network of free water or structural hydroxyl groups on the sample surface. The alteration occurs because chloride ions interact with or displace the surface hydroxyl groups, which weakens the hydroxyl vibrations.

After PS adsorbed chloride ions, the intensity of the Si–O–Si peak at 1032 cm^−1^ increased and the peak shape became sharper; the intensity of the Si–O–Si vibrational peak at 467 cm^−1^ increased; and the asymmetry of the C–O–C peak at 1425 cm^−1^ increased. Chloride ions are adsorbed onto silicate mineral surfaces through hydrogen bonding or electrostatic attraction, which induces ordering of the surface hydration layer and causes localized chemical perturbation of carbonate components. The siloxane framework as a whole did not exhibit any significant structural damage or amorphization (no significant peak shifts or broadening), in contrast to the chemical adsorption and structural rearrangement observed in samples modified by mechanical grinding during chloride ion adsorption [33,34].

It should be noted that absolute FTIR peak intensities are strongly influenced by sample preparation conditions. Therefore, to improve the reliability of spectral comparisons, the discussion was based on relative peak intensities normalized against a reference vibration band. In this study, the Si–O stretching vibration band was selected as an internal reference because of its structural stability. After normalization, the relative intensity variations of characteristic bands were analyzed to identify changes in functional groups. This approach reduces the influence of sample preparation conditions and enables a more reliable comparison of spectral features among different samples.

Figure 5b compares the FTIR spectra for PSM15 before and after chloride ion adsorption, with the latter denoted as PSM–Cl^−^. For PSM15, the spectral changes after chloride ion adsorption are more pronounced. The O-H stretching peaks at 3626 cm^−1^ and 3428 cm^−1^ become broader and stronger. The C–O–C peak at 1433 cm^−1^ becomes more intense. The Si–O–Si peak at 1037 cm^−1^ shifted to higher wavenumbers after grinding, indicating that the grinding process disrupted the silicoaluminate network; following chloride ion adsorption, the intensity of this peak increased, and the intensity of the 465 cm^−1^ peak was significantly enhanced [35]. This result may be associated with mechanical grinding, which destroyed mineral crystal structures in PS, including feldspar and quartz, generated abundant active sites, and further weakened the silicoaluminate framework [36]. During chloride ion penetration, chemical interactions occur between chloride ions and the activated surface. Some chloride ions enter the interlayer space through electrostatic attraction or ion exchange, thereby altering the chemical environment of the functional groups.

Furthermore, mechanical grinding modification promotes the formation of additional hydration products, specifically C–S–H gel, during chloride ion penetration. This observation is consistent with the SEM findings. The generation of C–S–H gel induces microstructural reorganization within the silicate network and enhances surface hydration. Consequently, the chemical interaction between chloride ions and the activated surface becomes more pronounced. The increased intensity in the hydroxyl region indicates a synergistic adsorption of water molecules and chloride ions, which further facilitates surface hydration.

After mechanical grinding modification, the number of active sites on the PS surface increases substantially. The grinding process enhances the dissolution and rearrangement of the silicoaluminate structure in PS and promotes surface hydration, thereby rendering the chemical interaction between chloride ions and the activated surface more pronounced.

### 2.5. Microstructural and Chemical Analysis (SEM–EDS)

**Figure 6:**
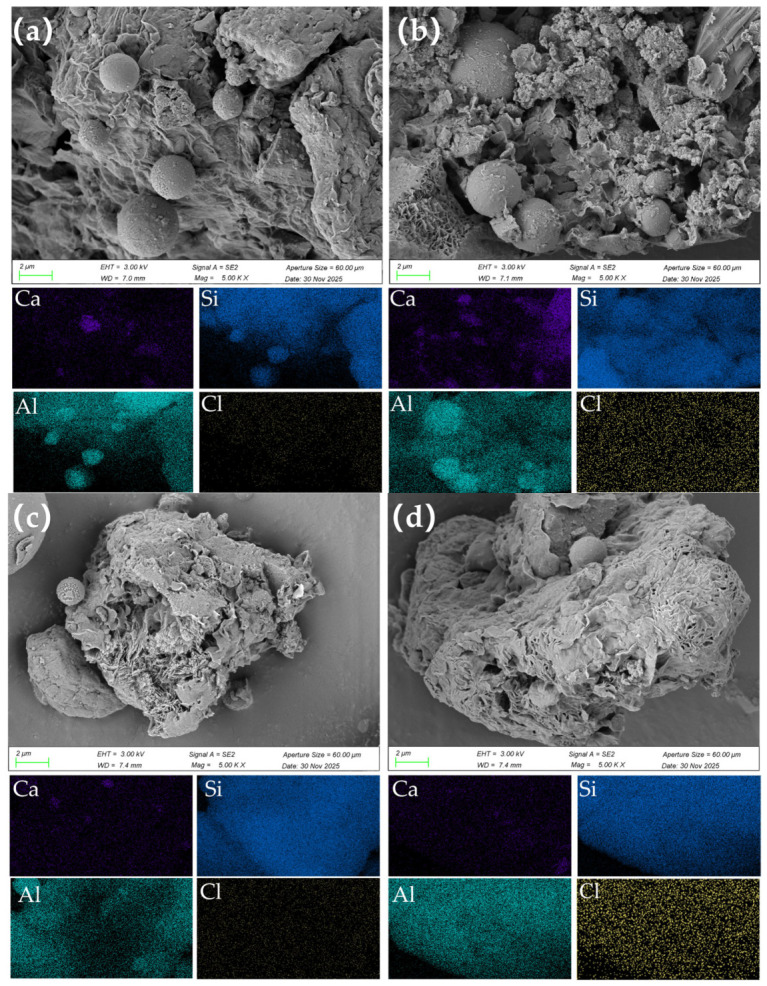
(**a**) SEM image of the PS sample before chloride ion adsorption, (**b**) SEM image of the PS sample after chloride ion adsorption, (**c**) SEM image of PSM15 before chloride ion adsorption, and (**d**) SEM image of PSM15 after chloride ion adsorption, showing the elemental surface scanning distributions of Ca, Si, Al, and Cl.

Figure 6a,b show a comparison of the microstructure of PS before and after chloride ion permeation. Figure 6a depicts an untreated specimen; its microstructure consists of arsenic sandstone particles serving as a skeleton, with interconnected pores and microcracks between the particles. The overall structure is relatively loose, and the particles are not tightly packed. Previous studies have indicated that among the hydration products of PS, C–S–H gel exists as a composite phase with a C/S (CaO/SiO_2_) molar ratio ranging from 0.25 to 2.3 [34,37,38,39]. EDS mapping revealed that Ca, Si, and Al were simultaneously distributed across the scanned region, indicating C–S–H gel formation, though in limited quantities. Due to the low reactivity of the source rock, the gel products are distributed sporadically and have failed to effectively fill the intergranular pores. Figure 6b depicts the morphology after chloride ion penetration. The microstructure undergoes pronounced changes: the original interconnected pores and microcracks become filled with abundant flocculent and clustered neo-formed products. Interparticle bonding appears denser, with no evident particle spalling or crack propagation. Chlorine is locally enriched, predominantly concentrated within the original pores and fracture fillings. These observations indicate that the specimen maintains good structural integrity in a chloride ion environment. Figure 6c,d show a comparison of the microstructures of the PSM15 specimens before and after chloride ion penetration. Figure 6c shows that after mechanical grinding, the particles exhibit a denser, multi-scale packing structure. In this structure, fine particles fill the spaces between the relatively larger particles, and the pores consist primarily of isolated small voids rather than interconnected cracks. Compared to Figure 6a, 15 min of mechanical grinding, through physical refinement and surface activation, transformed the microstructure of the arsenic sandstone specimen from loose and porous to dense and uniform, altering the chloride ion adsorption pattern from localized aggregation to uniform dispersion. After chloride ion adsorption, the layered structure in Figure 6d appears more continuous, with no independent clumps of clasts, indicating more thorough modification. Notably, distinct spherical particles can be clearly observed in the micromorphology. These spherical structures originated from the FA introduced during the modification process, and their morphology is consistent with the typical spherical shape of FA. The particles are embedded in the matrix, indicating a micro-filling effect. This effect contributes to an increase in active sites, thereby enhancing the adsorption performance of the material.

The literature confirms that N–A–S–H gel is a key hydration product in PS, composed of tetrahedral aluminates and alkali cations, forming gels with varying Al/Si ratios [40]. EDS mapping shows that Ca, Si, and Al are more extensively and continuously distributed, with notable enrichment in regions originally occupied by pores. This suggests that hydration products are uniformly deposited within pore channels and on particle surfaces. These elemental maps corroborate the formation of C–S–H and N–A-S–H gels [41,42,43]. Chlorine is homogeneously distributed in the maps without localized accumulation, demonstrating effective chloride ion adsorption. Previous studies [44,45] have established that the surface charge of C–S–H gel is governed primarily by its Ca/Si ratio. When the Ca/Si ratio is sufficiently high, the gel surface acquires a positive charge, thereby attracting chloride ions from the pore solution onto the C–S–H surface. The results indicate that mechanochemical activation of PS promotes the uniform generation and distribution of hydration products such as C–S–H and N–A–S–H gels. These products facilitate effective and homogeneous chloride ion adsorption through surface charge interactions; this provides microscopic evidence supporting the chlorine-fixing properties of modified PS.

### 2.6. Mechanism Analysis

Based on the integrated results of adsorption kinetics, removal efficiency evaluation, and microstructural characterization, the chloride-binding behavior of the modified PS cementitious system is governed by the synergistic contributions of mechanical activation, interfacial adsorption, retention within interlayer domains, and immobilization within hydration gel products. Among the investigated samples, PSM15 exhibited the most effective activation effect, which accounted for its superior chloride-binding capacity.

Mechanical activation is considered the dominant factor responsible for the enhanced adsorption performance of PSM15. Moderate grinding disintegrates coarse particles and agglomerates in PS. This process increases surface roughness and enlarges the effective contact area, thereby exposing additional potential adsorption sites. For framework minerals, such as quartz and feldspar, mechanical activation mainly occurs through particle size reduction and the generation of surface defects. For layered minerals such as montmorillonite, the shear forces generated during grinding may weaken some interlayer bonds, making interlayer entrances and edge sites more accessible. These observations are consistent with previous findings indicating that mechanical activation can enhance the reactivity of clay minerals [25,46,47,48].

After entering the material, chloride ions are initially adsorbed or retained on the external surfaces of particles, within open pores, and at the edge regions of layered minerals. Because the basal surfaces of montmorillonite generally carry a negative charge, substantial chloride uptake should not be simply ascribed to conventional interlayer ion exchange. A more plausible interpretation is that edge hydroxyl groups, defect sites, and hydration layer environments exposed by mechanical grinding provide localized adsorption sites for chloride ions [49]. Chloride ions can be retained in these regions through hydration layer rearrangement. However, the present results are insufficient to confirm the formation of abundant stable chemical bonds between chloride ions and the aluminosilicate framework [50].

From the perspective of interaction forces, the edge hydroxyl groups, defect sites, and hydration layer environments exposed by mechanical grinding provide localized adsorption sites for chloride ions. Moreover, the migration of hydrated chloride ions into interlayer entrance regions may disrupt the hydrogen bond network within surface hydration layers and involve O–H groups, thereby promoting localized chloride retention. Van der Waals interactions are relatively weak and make only a supplementary contribution to the overall physisorption process.

Gel products also contribute substantially to chloride immobilization. In an alkaline environment, the aluminosilicate components in PS can undergo partial dissolution and structural rearrangement, followed by reaction with reactive components, such as slag and fly ash, to form C–S–H and C–A–S–H gel phases [51]. Mechanical grinding enhances the surface reactivity of particles, thereby facilitating the formation and more uniform distribution of gel products on particle surfaces and within pore regions. These gel phases refine the pore structure and enhance matrix compactness. In parallel, they limit further chloride migration through surface adsorption and physical encapsulation. Previous studies have indicated that gel phases, such as C–S–H, can regulate chloride transport and immobilization through interfacial adsorption and pore confinement.

**Figure 7:**
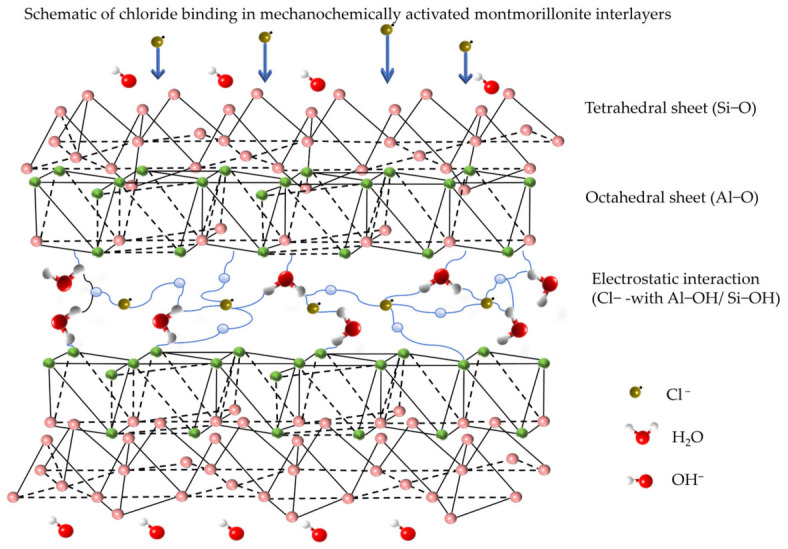
The mechanism of chloride ion adsorption on PSM15.

Thus, the mechanism underlying the enhanced chloride immobilization capacity of PSM15 can be summarized as a synergistic process involving mechanical activation, interfacial adsorption, interlayer retention, and gel immobilization. Mechanical grinding reduces particle size and exposes additional reactive surfaces, thereby increasing the contact opportunities between chloride ions and the material. The edge regions and defect sites of layered minerals provide localized retention sites for chloride ions. The formation of gel products further refines the pore structure and immobilizes chloride ions through adsorption and physical encapsulation. These synergistic effects jointly improve the chloride removal capacity of PSM15. Figure 7 illustrates the adsorption mechanism of chloride ions on PSM15.

Collectively, the chloride immobilization by modified PS should be described as a synergistic process dominated by surface adsorption, interlayer retention, and physical encapsulation by gel phases, accompanied by certain interfacial chemical interactions.

## 3. Conclusions

To achieve the resource utilization goal of converting PS into an efficient adsorbent material, the chloride adsorption performance, adsorption kinetic characteristics, phase composition, and microstructure of modified PS were systematically investigated. This investigation was conducted through chloride adsorption experiments, kinetic model fitting, and analyses employing XRD, FTIR, and SEM–EDS. The chloride uptake mechanism was further discussed. The principal conclusions are summarized as follows:

(1) Both mechanical grinding and high-temperature calcination enhanced the chloride adsorption performance of PS. Among the tested samples, PSM15 exhibited the highest adsorption capacity, reaching equilibrium within 240 min and achieving a chloride removal efficiency of 33.3%, which was markedly higher than those of raw PS (23.6%) and PSC600 (30.1%). The adsorption kinetics of chloride ions onto modified PS were well described by the pseudo-second-order model, with a maximum correlation coefficient of 0.988. The results indicate that the chloride ion adsorption process on modified PS can be attributed, at least in part, to surface chemical interactions.
(2) XRD analysis showed that the modification treatment did not alter the main crystalline phases of PS. However, mechanical grinding markedly enhanced the chloride adsorption capacity. This improvement was attributed to crystallite refinement, exfoliation of the layered montmorillonite structure, and exposure of surface active sites. In the PSM15 sample, the low-angle characteristic peak of montmorillonite became much stronger and more distinct, providing direct evidence of mechanochemical exfoliation.
(3) FTIR and SEM-EDS characterization further indicated that, after modification, the chemical environment of characteristic functional groups such as Si–O–Si and O–H changed significantly. Mechanical grinding transformed the microstructure of the sample from loose and porous to dense and homogeneous. It also promoted the uniform formation of C–S–H and N–A–S–H gels. These gel phases efficiently and stably immobilized chloride ions through physical encapsulation.

Overall, modified PS-based cementitious material (PSM15) represents a low-cost and eco-friendly adsorbent material with considerable promise for applications such as the treatment of chloride-containing wastewater. This study provides a viable pathway for transforming PS from an environmental hazard into a highly efficient adsorbent material and offers a novel adsorbent for the remediation of chloride-contaminated water bodies. The potential recyclability of the material is suggested by its structural stability; however, further cyclic adsorption–desorption studies are required for quantitative evaluation.

## 4. Materials and Methods

### 4.1. Materials

**Figure 8:**
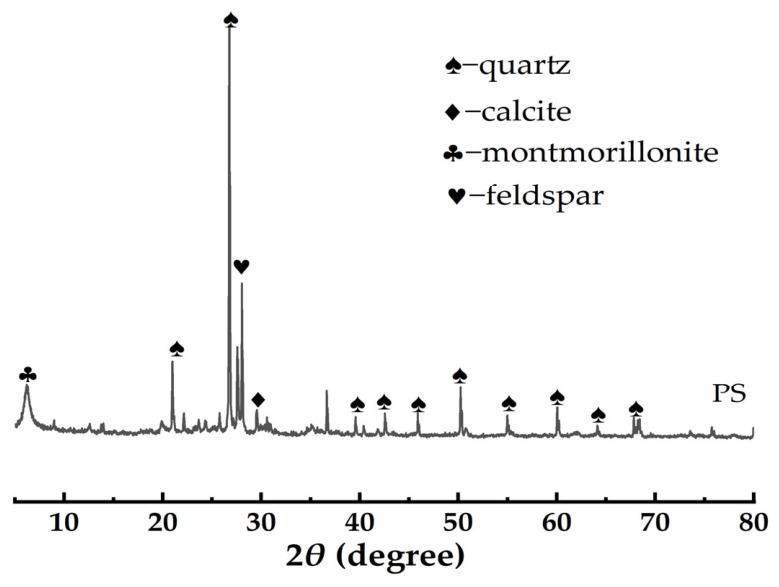
XRD analysis of PS.

**Figure 9:**
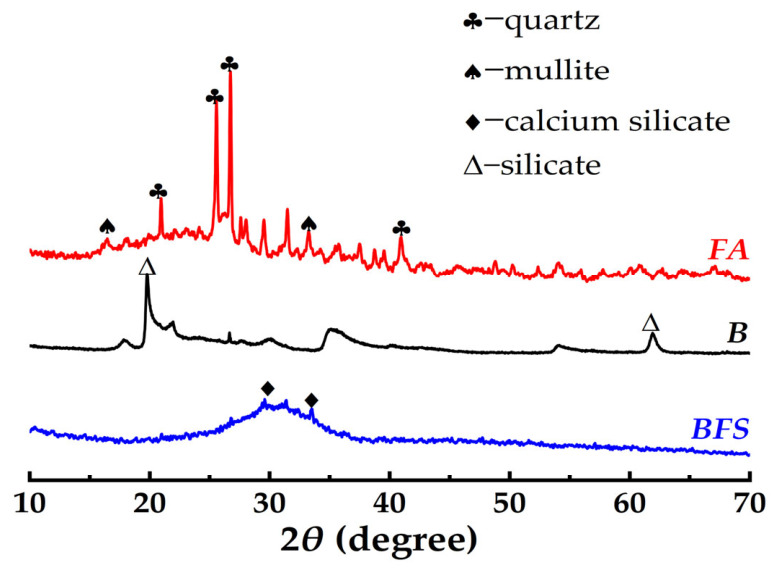
XRD analysis of additives.

The PS used in the experiments was collected from Ordos City, Inner Mongolia (China). The raw material was air-dried, crushed, and passed through a 0.6 mm square mesh sieve prior to use. Bentonite (B) was supplied by Guzhang County Shanlin Shiyu Mineral Products Co., Ltd. (Anhui, China). S95-grade ground granulated blast furnace slag (BFS) and Class II fly ash (FA) were both sourced from Zhengzhou City (Zhengzhou, China). The chemical composition and mineral composition of PS, B, BFS and FA were analyzed by X–ray fluorescence (XRF) and X–ray diffraction (XRD). The test results are shown in Table 3 and Figure 8 and Figure 9. Sodium chloride (NaCl) of analytical grade, obtained from Sinopharm Chemical Reagent Co., Ltd. (Nanjing, China), was used as the solid chloride ion source. The alkali activator solution was prepared from sodium hydroxide and sodium silicate with a modulus of 2.0 (molar ratio of SiO_2_ to Na_2_O). All chemical reagents were procured from Tianjin Kemeiou Chemical Reagent Co., Ltd. (Tianjin, China). To avoid interference from extraneous ions, deionized water was used throughout the experimental procedures.

### 4.2. Mechanically Activated PS

Raw PS was loaded into a high-temperature box-type resistance furnace (Shanghai Jingzhao Machinery Equipment Co., Ltd., Shanghai, China), and separate batches were heated to 400 °C, 500 °C, and 600 °C at a constant 5 °C/min ramp rate. The material was held at each target temperature for 2 h. After the holding period, the furnace power was turned off, and the samples were allowed to cool naturally to room temperature. The resulting calcined specimens were stored in airtight sealed bags for subsequent use, with each batch fixed at 100 g. In a separate procedure, additional raw PS was subjected to dry grinding using a YXQM–8 vertical planetary ball mill (Feishi Scientific Instruments Co., Ltd., Beijing, China). Zirconia (ZrO_2_) balls with a diameter ratio of 6 mm:2 mm = 6:4 were employed as the grinding media. The main shaft rotational speed was set to 340 r/min. Samples were ground for 15 min, 30 min, and 45 min, respectively, and then collected and preserved in airtight sealed bags for subsequent use.

### 4.3. Adsorption Test

Batch adsorption experiments were conducted at 25 °C (room temperature) and pH = 7. In each experiment, 0.1 g of treated PS and 50 mL of sodium chloride solution with a chloride ion concentration of 0.01 mol/L (355 mg/L) were added to a 100 mL centrifuge tube. The tubes were transferred to a thermostatic shaker (Model THZ-92 C) and agitated at 200 r/min. At predetermined time intervals (1, 3, 5, 10, 30, 60, 120, 240, 480, 720, and 1440 min), individual tubes were removed and centrifuged at 3500 r/min for 15 min. The supernatant was collected, passed through a 0.45 μm Whatman microporous membrane, and analyzed for chloride ion concentration using ICP-OES (PE, USA). A time-zero measurement, obtained by sampling immediately after the addition of PS, was also performed to correct for any initial changes in concentration. All experiments were conducted in triplicate, and blank control groups were included. The reported values are the arithmetic means of the replicate measurements.

Based on the measured chloride ion concentrations, the adsorption capacity *qₜ* (mg/g) was calculated using Equation (1):

(1) $$q_{t}=\frac{C_{0}-C_{i}}{m}\;\times \;V$$

where *qₜ* is the adsorption capacity per unit mass of the sample (mg/g); C_0_ and *C_i_* denote the initial ion concentration and the ion concentration in the solution after adsorption (mg/L), respectively; V represents the volume of the added NaCl solution (mL); and m is the mass of the PS sample (g).

The removal efficiency of chloride ions (*R*) by the specimen was calculated using Equation (2):

(2) $$R\;=\;\frac{C_{0}-C_{e}}{C_{0}}\;\times \;100\%$$

where C_0_ is the initial ion concentration in the solution (mg/L) and *Cₑ* is the equilibrium concentration of residual ions in the solution (mg/L).

Assuming that the measured concentration equals the surface concentration, the pseudo-first-order and pseudo-second-order kinetic models (Equations (3) and (4)) were employed to fit the experimental adsorption kinetic data.

(3) $$ln(q_{e}-q_{t})=lnq_{e}-(k_{1}/2.303)t$$

(4) $$\frac{t}{q_{t}}=\frac{1}{k_{2}{q_{e}}^{2}}+\frac{t}{q_{e}}$$

where *qₑ* is the adsorption capacity at equilibrium (mg/g); *qₜ* is the adsorption capacity at time t (mg/g); *k_1_* is the pseudo-first-order rate constant (min^−1^); and *k_2_* is the pseudo-second-order adsorption rate constant (g/(mg·min)).

To further analyze the adsorption kinetics and diffusion mechanisms, the experimental kinetic data were fitted using the intramolecular diffusion model. This model elucidates the diffusion characteristics and the underlying mechanism governing the adsorption process, as expressed in Equation (5).

(5) $$q_{t}={\;K}_{\mathrm{int}}t^{0.5}+C$$

where *K_int_* (mg/g/min^0.5^) and *q_t_* (mg/g) represent the intramolecular diffusion rate constant and the adsorption capacity, respectively, and C is the intercept, which relates to the thickness of the boundary layer.

In addition to the pseudo-first-order and pseudo-second-order kinetic models and the intramolecular diffusion model, the Elovich model was introduced to further evaluate the chemisorption behavior of chloride ions on the modified PS adsorbents. The Elovich model is generally suitable for adsorption on energetically heterogeneous surfaces and can provide additional information on the initial adsorption rate and surface activation energy. The linear form of the Elovich model is expressed in Equation (6):

(6) $$q_{t}\;=\;\frac{1}{\beta }\;\ln(\alpha \beta )\;+\;\frac{1}{\beta }\ln\;t$$

where *q_t_* is the adsorption capacity at time t, mg/g; *α* is the initial adsorption rate constant, mg/(g min); and *β* is the Elovich constant related to surface coverage and the activation energy of chemisorption (g/mg).

### 4.4. Preparation of Modified PS Test Specimens and Adsorption Experiments

**Figure 10:**
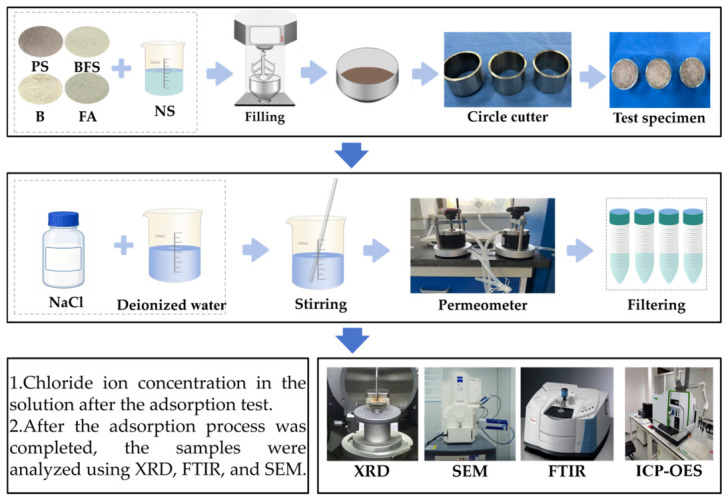
The flowchart for preparing the modified PS samples and their adsorption of chloride ions.

The solid mixture (modified PS and additives) and the modifying agent were weighed according to the specified proportions (PS: 73%, B: 5%, BFS: 5%, FA: 5%). The modifying solution consisted of sodium silicate and water at a ratio of 80:20. After weighing, the mixture was pre-stirred using a magnetic stirrer to achieve uniformity. This step allowed the liquid phase to initially wet the solid particles and reduce local agglomeration. Mixing was initiated at low speed for 30 s while the modifying agent was added, followed by high-speed mixing for 180 s to ensure thorough blending. To minimize batch-to-batch variation, identical mixing times and mixing protocols were adopted for all specimen groups. After mixing, the mixture was compacted into cutting rings in layers. During compaction, the target dry density was controlled at 1.5 g/cm^3^ so that all specimens started from a relatively consistent initial compacted state. The prepared specimens were covered with plastic film and cured in a standard curing room for 7 d. Subsequently, ion adsorption tests were conducted. The specimens were placed in a permeameter, and the chloride ion solution that percolated through the PS specimens was collected. The chloride ion concentration was measured after each collection of 50 mL of the effluent. The preparation of modified PS and the adsorption process for chloride ions are shown in Figure 10.

### 4.5. Characterization of Modified PS

#### 4.5.1. XRD

XRD analysis of the sample was performed using a Bruker D8 Advance X–ray diffractometer (Brook Technology Inc., USA, located in Billericay, MA, USA), with particular attention given to the mineralogical composition of PS. Diffraction data were collected with Cu Kα radiation (*λ* = 1.54 Å), while the X–ray generator operated at 40 kV and 30 mA. The scan covered a 2*θ* range from 5° to 80°, with a 5°/min scanning rate and a 0.02° step size.

#### 4.5.2. FTIR

FTIR was performed using a Spectrum GX FTIR spectrometer (Shanghai Precision Scientific Instrument Co., Ltd., Shanghai, China). The spectra were collected over the range of 4000–500 cm^−1^ using the pellet method. Prior to analysis, the samples were ground, passed through a 45 μm sieve, and subsequently vacuum-dried at 40 °C for 12 h. To ensure the reproducibility of FTIR measurements, all samples were prepared following a strictly controlled and identical protocol. Each sample was dried under the same conditions, finely ground, and mixed with KBr at a fixed mass ratio before pellet pressing. The pressure applied during pellet formation and the thickness of the pellets were maintained as consistently as possible. In addition, at least three parallel measurements were conducted for each sample to verify spectral consistency.

#### 4.5.3. SEM–EDS

Prior to SEM observation, the samples were vacuum-dried. Selected representative fragments were then coated with gold to improve their surface conductivity. The surface morphology and microstructural features of the samples were examined using a Sigma 300 scanning electron microscope manufactured by ZEISS (German Carl Zeiss Group, Oberkochen, Germany).

#### 4.5.4. BET Specific Surface Area Analysis

The BET (Beiside Instrument Technology Co., Ltd., Beijing, China) specific surface areas of PS, PSM15, PSC400, PSC500, and PSC600 were measured using a nitrogen adsorption analyzer. Prior to analysis, about 0.5 g of each powdered sample was dried at 60 °C for 12 h, followed by vacuum degassing at 105 °C for 6 h. Nitrogen adsorption–desorption isotherms were then recorded at 77 K. The BET surface area was calculated from the adsorption branch over a relative pressure (P/P_0_) range of 0.05–0.30. All measurements were performed in triplicate, and the mean value was used for subsequent analysis.

## Figures and Tables

**Table 1:** Kinetic parameters for chloride adsorption on different PS-based adsorbents (pseudo-first-order, pseudo-second-order, Weber–Morris and Elovich models).

Kinetic Model	Parameter	PS	PSC600	PSC500	PSC400	PSM45	PSM30	PSM15
Pseudo-first-order	k_1_ (/min)	0.16	0.07	0.17	0.14	0.15	0.13	0.16
	q_e,cal_ (mg/g)	7.2	6.94	7.46	6.72	6.96	6.87	7.25
	R^2^	0.938	0.978	0.952	0.898	0.912	0.929	0.897
Pseudo-second-order	k_2_ × 10^−4^ (g/mg/min)	1.47	4.46	1.28	1.13	6.24	4.09	5.39
	h (mg/g/min)	1.82	0.97	0.27	0.26	1.25	0.91	1.33
	R^2^	0.975	0.988	0.983	0.974	0.929	0.976	0.943
Weber–Morris model	K_1,1_ (mg/g/min^0.5^)	1.63	2.39	2.54	2.49	1.84	2.21	2.18
	C_1,1_ (mg/g)	9.29	1.8	5.27	5.36	7.98	3.16	7.38
	R^2^_1,1_	0.89	0.91	0.915	0.849	0.92	0.83	0.92
	K_1,2_ (mg/g/min^0.5^)	0.12	0.02	0.07	0.06	0.14	0.02	0.13
	C_1,2_ (mg/g)	30.21	44.06	41.67	42.62	38.42	46.04	43.73
	R^2^_1,2_	0.94	0.33	0.892	0.493	0.951	0.54	0.914
Elovich	α (mg/g/min)	15.45	15.48	12.89	12.53	20.11	18.54	21.59
	β (g/mg)	0.08	0.05	0.05	0.05	0.06	0.06	0.06
	R^2^	0.942	0.956	0.919	0.897	0.919	0.849	0.942

**Table 2:** Comparison of chloride ion adsorption performance between PSM15 in this study and previously reported adsorbents.

Literature	Adsorbent	Main Test Conditions	Adsorption Performance	Main Adsorption Mechanism	Material Characteristics
Yang et al. [17]	CaFeAl LDHs	Simulated concrete pore solution and artificial seawater system	The maximum adsorption capacity is 3.18 mmol/g, which is approximately 112.7 mg/g	Surface adsorption and ion exchange	Suitable for cement-based systems, but the synthesis process is rather complex
Xu et al. [20]	CaMnFe LDHs	Adsorption in aqueous solution, Langmuir model fitting	Maximum adsorption capacity: 144.01 mg/g	Oxygen-containing functional groups adsorption and ion exchange	Strong adsorption capacity, which can enhance the chloride ion curing ability of the cement slurry
Zhang et al. [28]	UHLA System	NaAlO_2_ and CaO composite system, 25 °C; 60 min	The maximum adsorption capacity is 63.50 mg/g; at low concentrations, 93.3% can be removed	Aluminate reaction products are combined and precipitated for fixation	The reaction is relatively fast, but the requirements for the composition of the system and the control of reaction conditions are quite high
This study	PSM15	NaCl solution, 25 ± 1 °C; mechanical grinding for 15 min	Adsorption capacity of about 48 mg/g; removal efficiency of 33.3%	Surface interaction, interlayer fixation, and encapsulation by C–S–H and N–A–S–H gels	Abundant natural source, simple preparation, low cost, and environmentally friendly feature

**Table 3:** Chemical composition of PS and additives (wt%).

Item	SiO_2_	Al_2_O_3_	CaO	Na_2_O	K_2_O	MgO	Fe_2_O_3_	TiO_2_
PS	65.20	15.65	2.02	1.26	3.97	3.86	6.82	0.68
FA	42.26	34.61	10.81	0.1	0.74	0.87	3.26	2.20
B	64.98	15.87	2.23	0.62	0.53	5.32	2.37	0.15
BFS	30.36	9.97	44.36	0.53	0.74	7.59	0.54	2.11

## Data Availability

Data will be made available on request.
